# Functional and morphologic study of retinal hypoperfusion injury induced by bilateral common carotid artery occlusion in rats

**DOI:** 10.1038/s41598-018-36400-5

**Published:** 2019-01-14

**Authors:** Yali Qin, Meiqi Ji, Tingting Deng, Dan Luo, Yingxin Zi, Lin Pan, Zhijun Wang, Ming Jin

**Affiliations:** 1 Beijing University of Chinese Medicine, Beijing, 100029 China; 2 Department of Ophthalmology, China-Japan Friendship Hospital, Beijing, 100029 China; 3 Clinical Medical Research Institute, China-Japan Friendship Hospital, Beijing, 100029 China

## Abstract

Retinal hypoperfusion injury is the pathophysiologic basis of ocular ischemic syndrome (OIS) which often leads to severe visual loss. In this study, we aimed to establish a rat model of retinal chronic hypoperfusion by bilateral common carotid artery occlusion (BCCAO) and observe changes in the retinal function and morphology. We found that model rats showed retinal arteriosclerosis, slight dilated retinal vein, small hemangiomas, hemorrhages, vascular segmental filling, and nonperfused areas after 2 weeks of BCCAO. In the model rats, the retinal circulation time was significantly prolonged by fluorescein fundus angiography (FFA), the latency of a and b waves was delayed and the amplitude was decreased significantly at each time point by electroretinogram (ERG), and the perfusion of the eyes continued to reduced. Morphologic and ultrastructural changes covered that the retinal ganglion cells (RGCs) presented obvious apoptosis and the thickness in the retinal layers were significantly thinner. Collectively, these findings suggested that BCCAO induced retinal hypoperfusion injury in the model rats, thus providing an ideal animal model for the study of OIS.

## Introduction

Retinal hypoperfusion injury is an ocular circulatory disorder caused by carotid artery stenosis or occlusion. It belongs to the category of ocular ischemic syndrome and is often associated with ischemic encephalopathy. This disorder has now become a subject of cross-disciplinary study involving both ophthalmology and neurology^1^. The mortality rate among OIS patients within 5 years of onset is as high as 40%^2^. Cardiovascular disease is the leading cause of such deaths, accounting for about 66% of them^3^. Retinal hypoperfusion injury is the pathophysiologic basis of many ocular posterior ischemia diseases, such as retinal artery occlusion, retinal vein occlusion, ischemic optic neuropathy, and neovascular glaucoma^4^. It has been reported that 67% of these patients gradually lose their eyesight within weeks or months^5^. Because of the lack of specificity of symptoms and the inconsistent level of ischemia in the early onset of the OIS, the risk of severe visual loss and even blindness in later stages has become a ophthalmologic problem. Therefore the establishment of an ideal experimental model of ocular ischemia and exploratory study of its characteristics and mechanisms are of significant importance for the clinical diagnosis and treatment of OIS.

Currently extensive experimental studies on acute ocular ischemia-reperfusion are under way, applying models with high intraocular pressure, optic nerve bundle ligation or clamping, blood vessel ligation, photodynamic induction, or vitreous cavity injection^6–11^. However, more work still needs to be done in the experimental study of ocular chronic ischemia. Bilateral common carotid artery occlusion (BCCAO) is a method of inducing chronic ocular ischemia; it has gradually been accepted in recent years because its pathological process mimiced clinical OIS caused by carotid artery stenosis or obstruction^12,13^.

In our study, rats, whose retinas are similar to those of humans anatomically, were selected to serve as the animal model. Chronic retinal hypoperfusion injury models were established by bilateral common carotid artery occlusion. The retinal functional and morphologic changes were systematically compared and studied during different observation periods and at different sites.

## Results

### Survival records of rats

In our study, all the sham-group rats survived for experimental pupropses. In the BCCAO group, the rats survived within first week. However, one BCCAO rat died at the second week and two BCCAO rats died at the fourth week. The other rats in the BCCAO group survived for longer than 4 weeks.

### Slowed tail - retinal circulation time after BCCAO

**Figure 1:**
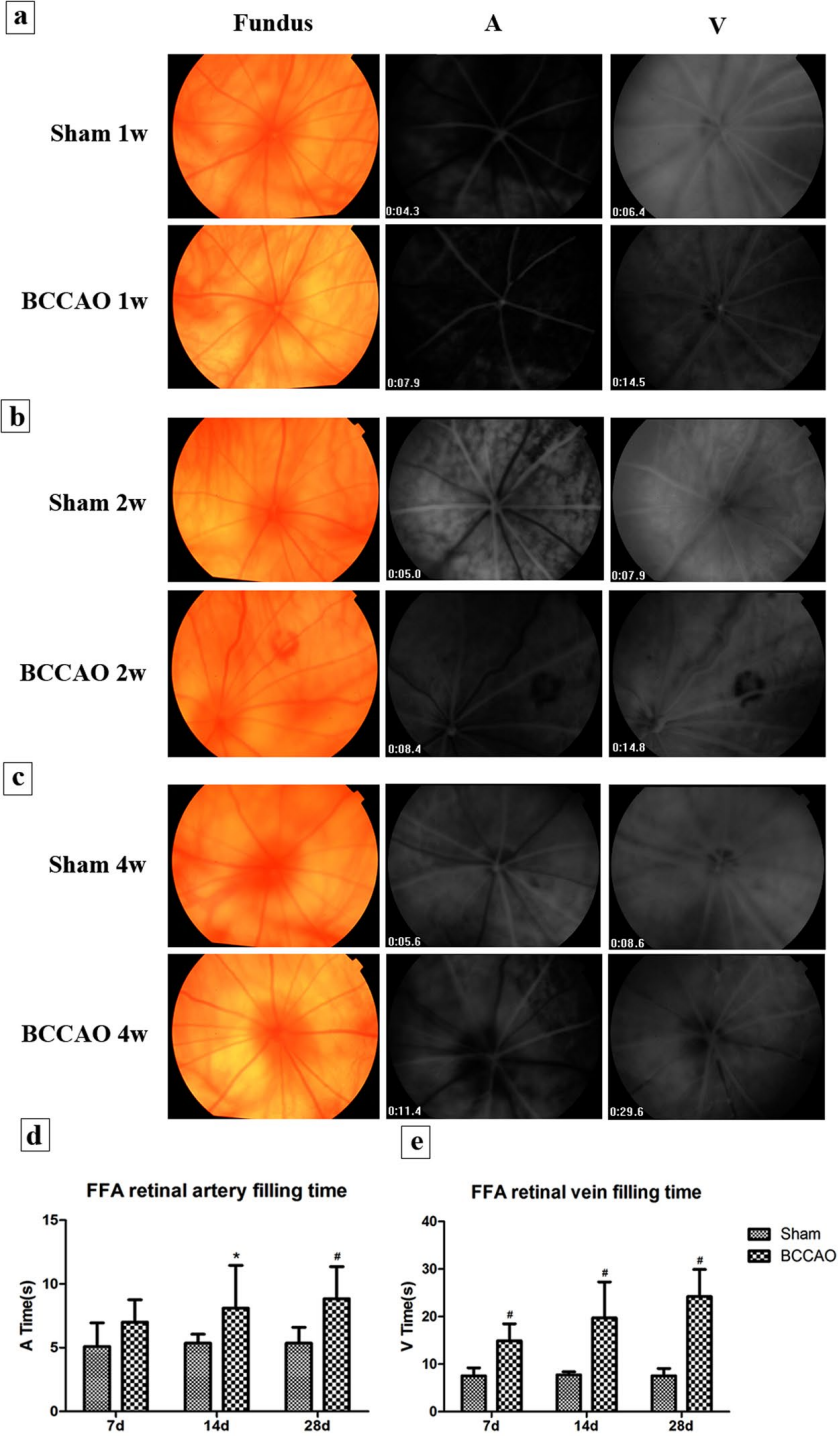
Retinal fundus photograph and FFA of rats. The operational requirements: Scope of photo was 30° field and the exposure level was at 50. (**a**) The tail - retinal circulation time at 1w. (**b**) The tail - retinal circulation time at 2w. (**c**) The tail - retinal circulation time at 4w. (**d**) The comparison of retinal arterial filling time in different groups. (**e**) The comparison of retinal venous filling time in different groups (n = 8 to 10, ^*^P < 0.05 compared with sham group, ^#^*P* < 0.01 compared with sham group).

In the sham group, the rich retinal blood flow showed high fluorescence, and the retinal blood vessels filled very rapidly. However, the model group (BCCAO) showed retinal arteriosclerosis, slight dilated retinal vein. The retinal blood circulation time began to slow down at 1 week (Fig. 1a), small hemangiomas and hemorrhages were found at part of the retina at 2 weeks (Fig. 1b), and segmental filling of the vessels and capillary non-perfusion zones were found at the 4 weeks of subgroup (Fig. 1c). Retinal arterial filling time is denoted by A, and retinal venous filling time is represented by V. Compared with rats in sham group (A = 5.09 ± 1.85 s, 5.36 ± 0.69 s, 5.36 ± 1.23 s and V = 7.56 ± 1.67 s, 7.74 ± 0.65 s, 7.56 ± 1.53 s), the retinal arterial filling time had no obvious change after BCCAO of 1 week rats (A = 7.01 ± 1.74 s, *P* > 0.05, Fig. 1d); but they were prolonged at the 2 and 4 weeks of subgroups (A = 8.11 ± 3.35 s, 8.84 ± 2.51 s, *P* < 0.05). Retinal venous filling time was significantly prolonged at each time subgroup (V = 14.91 ± 3.57 s, 19.73 ± 7.55 s, 24.21 ± 5.70 s, *P* < 0.01, Fig. 1e), and some rats’ retinal branch veins were not fully filled even at 1 minute.

### Changes of latency and amplitude of a- and b-waves in scotopic ERG

**Figure 2:**
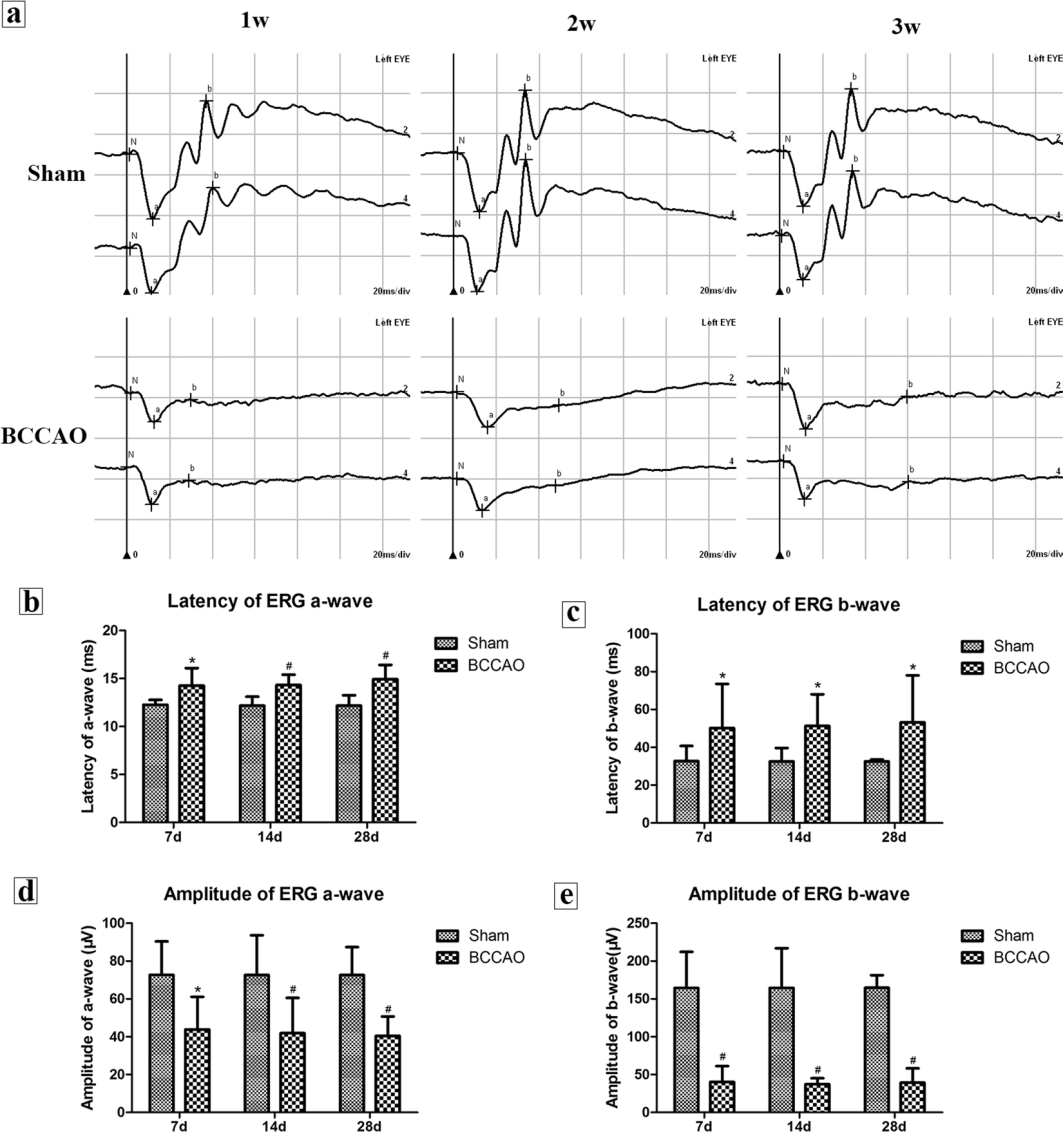
Retinal scotopic ERG of rats. (**a**) Changes of latency and amplitude of a- and b-waves at 1,2 and 4 weeks. (**b**) The latency of the a-wave in different groups. (**c**) The latency of the b-wave in different groups. (**d**) The amplitude of the a-wave in different groups. (**e**) The amplitude of the b-wave in different groups (n = 8 to 10, ^*^P < 0.05 compared with sham group, ^#^*P* < 0.01 compared with sham group).

The latency (a-waves were about 12 ms, b-waves were about 32 ms) and amplitude (a-waves were about 72 μV, b-waves were about 164 μV) in the sham rats at all time subgroups were very stable (Fig. 2a). With the prolongation of hypoperfusion time, the latency of the a- and b-waves in the model rats showed a trend toward increasing delay (Fig. 2a). Statistics showed that compared with sham group, latency of a-waves (from 14.25 ms to 14.92 ms, Fig. 2b) and b-waves (from 50.17 ms to 53.17 ms, Fig. 2c) both severely extended in the model rats at 1, 2, and 4 weeks (*P* < 0.05). In the model rats, the amplitudes of the a-waves (from 43.91μV to 40.44 μV, Fig. 2d) and b-waves (from 40.32 μV to 37.14 μV, Fig. 2e) decreased significantly at 1, 2, and 4 weeks (*P* < 0.05). The amplitude of the b-wave in model rats decreased most significantly at 2 weeks and did not return to the baseline level. Although it increased slightly at 4 weeks after BCCAO, it reached only 1/5 to 1/4 of the sham rat.

### Changes in blood perfusion of the eyes’ surface

**Figure 3:**
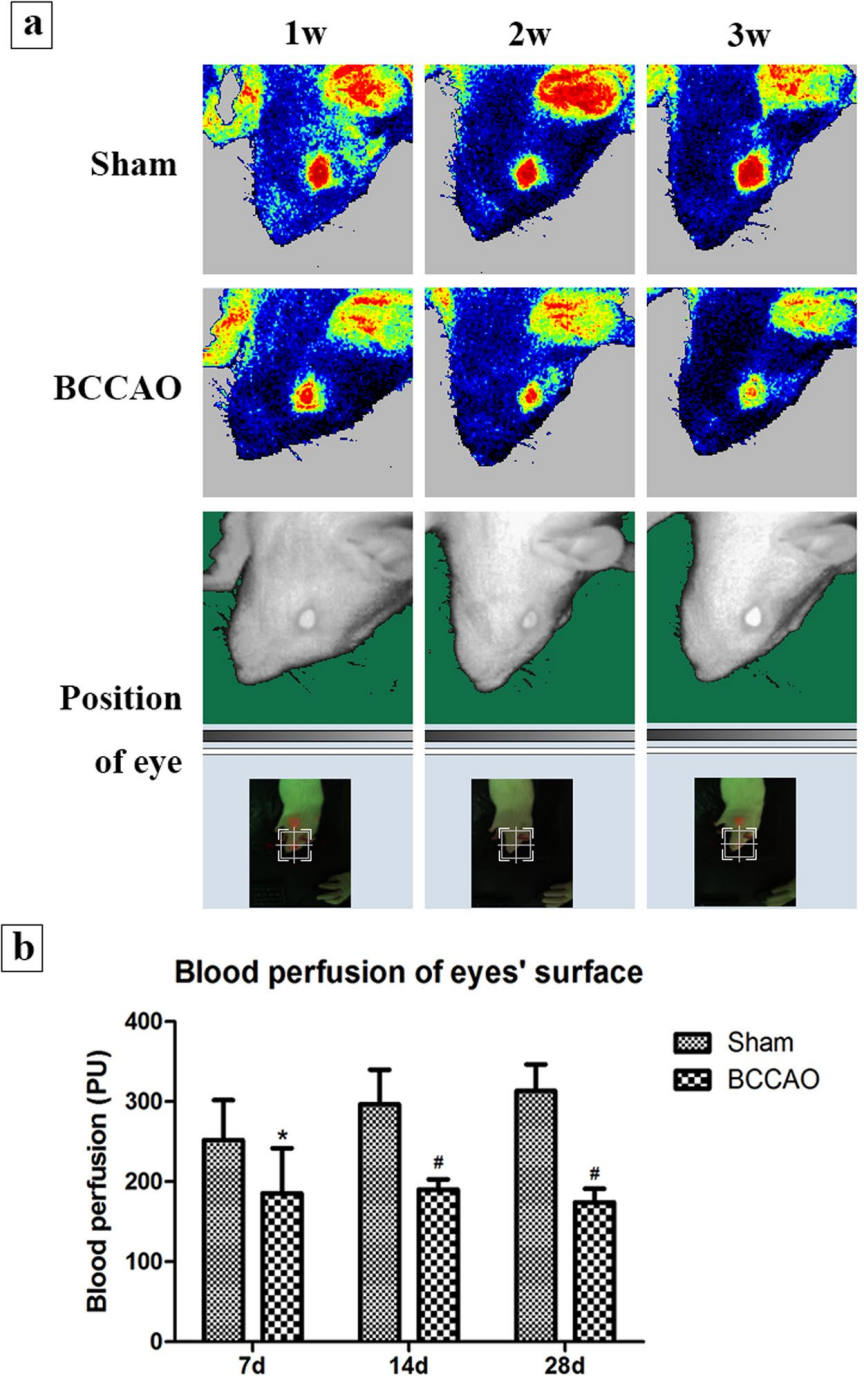
The blood perfusion of rats’ eyes. The operational requirements: constant temperature (23.0 + 1.0 °C), relative humidity (55.0 + 5.0%), no direct sunlight, no obvious air convection and no noise interference. (**a**) The laser speckle blood flow imager of the eyes’ surface in different groups. (**b**) Changes in blood perfusion of the eyes’ surface in different groups (n = 8 to 10, ^*^P < 0.05 compared with sham group, ^#^*P* < 0.01 compared with sham group).

The average of blood flow perfusion of the binocular surfaces was selected as the final result of each rat for data statistics. There were fluctuations of blood perfusion in sham rats at different time subgroups as the growing weight over time, but the differences were not significant. Compared with sham group (from 251.98 PU to 313.11 PU), the blood perfusion in the model rats decreased significantly at the 1 and 2 weeks of subgroups (from 185.51 PU to 190.21 PU, *P* < 0.05). At the 4-week subgroup, the average blood perfusion of the model rats was lowest than in the sham group and the other time model subgroups (174.08 PU, *P* < 0.01) (Fig. 3a,b).

### Retinal histology and morphologic and changes of thickness

**Figure 4:**
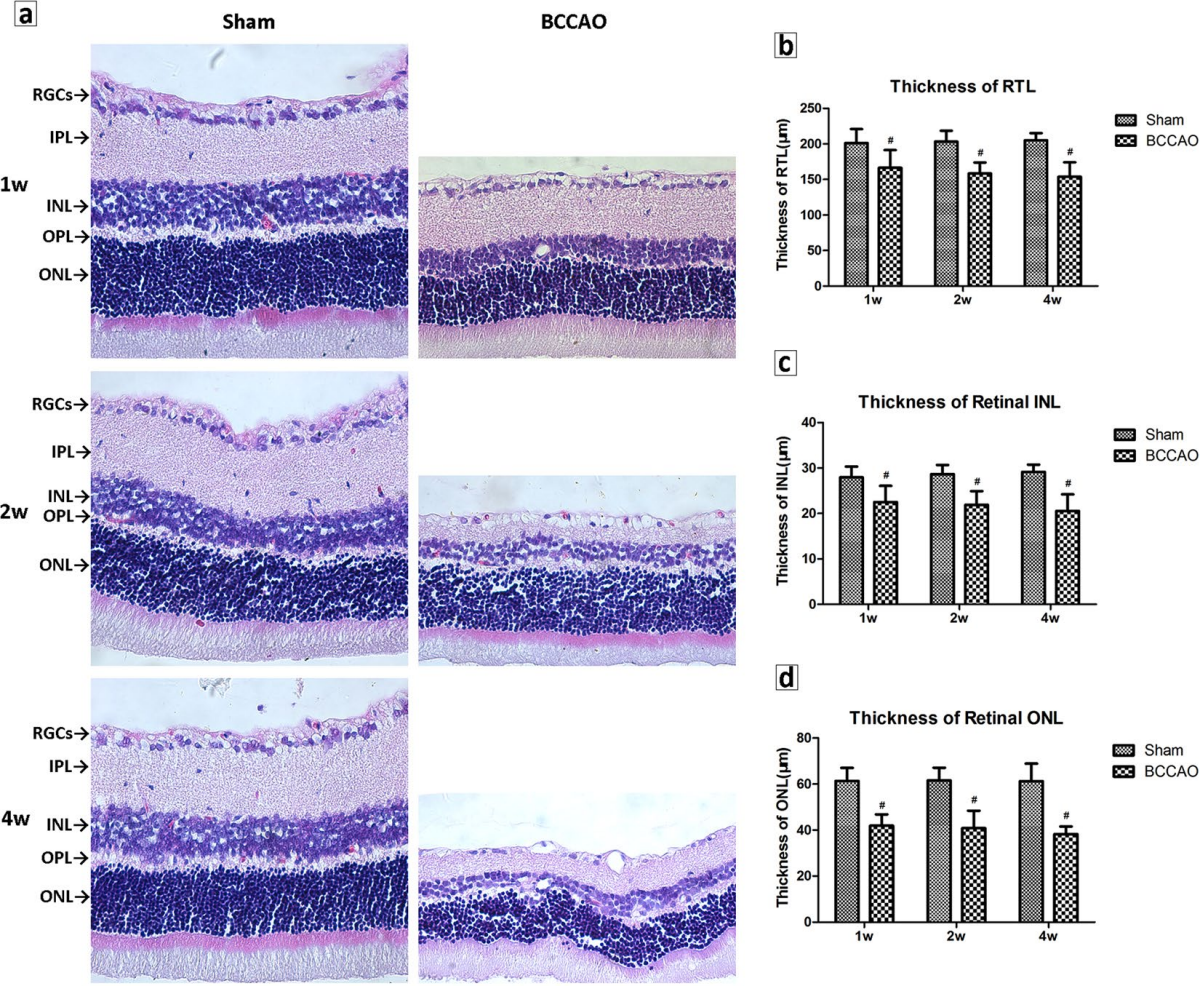
Retinal histology and morphology in HE. The magnification of retinal images were 10 times (eyepiece) *40 times (objective) with a scale bar of 50 microns (50 μm). (**a**) Retinal morphologic of different groups at 1, 2 and 4 weeks. (**b**) The thickness of the RTL in different groups. (**c**) The thickness of the INL in different groups. (**d**) The thickness of the ONL in different groups (n = 8 to 10, ^#^*P* < 0.01 compared with sham group).

Under the optical microscope, the sham-operated rats had dense, well-arranged, and clearly demarcated tissues in each layer of the retina. The retinal ganglion cells (RGCs) were arranged in a monolayer, with larger nuclei and darker staining. The nuclei of the cells of the inner nuclear layer were larger and stained darker. The outer nuclear layer was thick and the cells were compact. However, the rats’ retina in the model group had disordered arrangement of retinal layers, a reduced number of RGCs, nuclear pyknosis, chromatin marginalization, necrosis and dissolution of some cells, with changes of vacuolization. There was marked thinning of the inner and outer nuclear layers. The staining was lighter and vacuolar degeneration of the cells was evident. The inner plexiform layer (IPL), outer plexiform layer (OPL) and external limiting membrane became thin. Overall the structure of the photoreceptor layer was loose (Fig. 4a). The retinal total layer (RTL) is defined as the distance between the inner membrane and the outer membrane. Compared with the sham group (RTL were about 201.08 to 205.18 μm), the thickness of the RTL was significantly lower in model group rats (RTL were 166.30 μm, 158.49 μm, and 153.83μm at 1, 2 and 4 week, *P* < 0.01, Fig. 4b). In the early stage, the atrophy of inner nuclear layer (INL) appeared and then the outer nuclear layer (ONL) began to shrink. Both of the thickness in the INL and the ONL were significantly thinner (*P* < 0.01, Fig. 4c,d) at each time subgroup.

### Changes in ultrastructure of retinal nerve cells

**Figure 5:**
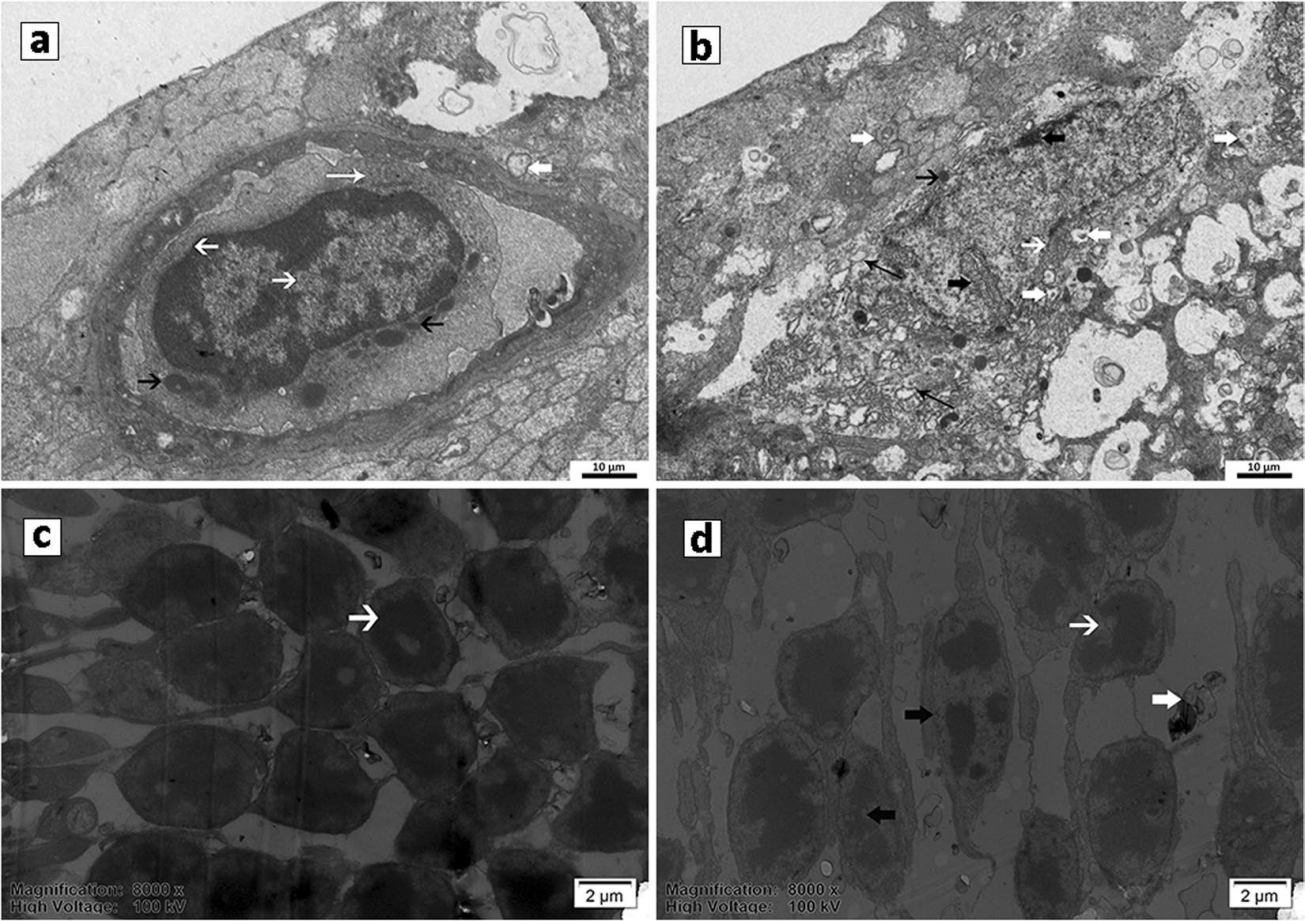
The Changes in ultrastructure of retinal nerve cells. (**a**) RGC in sham rats contained large and unbroken nuclei and intact nuclear membranes (thin short white arrow), and rich mitochondria (thin short black arrow) and endoplasmic reticulum (long white arrow). (**b**) RGC in BCCAO rats showed serious karyopyknosis, chromatic agglutination (thick short black arrow), decreased or swelling mitochondria (thin short black arrow) and vacuolization of endoplasmic reticulum (long black arrow), surrounded with a large number of proliferating microglial cells (thick short white arrow). (**c**) ONL cells in sham rats were neatly and tightly arranged, with unbroken nuclei (short white arrow). (**d**) ONL cells in BCCAO rats were with shrunken and invaginated nuclear membranes (short white arrow), and the chromatin was uneven density (short black arrow).

Under the transmission electron microscope,the sham rats showed clear structures of the RGCs, with large and unbroken nuclei, intact nuclear membranes, rich mitochondria and endoplasmic reticulum (Fig. 5a). The cells of ONL were closely connected and uniform chromatin density. The outer segments of the photoreceptors were neatly and tightly arranged (Fig. 5c). At the 4-week subgroup after BCCAO, the RGCs of the model rats were disordered and seriously damaged: karyopyknosis, chromatic agglutination and decreased or swelling organelles. They were surrounded by a large number of proliferating microglial cells (Fig. 5b). The ONL cell nuclei were of different sizes, the nuclear membranes were shrunken and invaginated, and the chromatin was uneven density. The outer segments of the photoreceptors were loose, fractured, and even dissolved (Fig. 5d).

## Discussion

The ophthalmic artery is one of the main branches of the internal carotid artery going into the brain. Carotid artery stenosis will reduce the perfusion pressure of the central retinal artery by about 50%^3^. When the carotid artery is severely stenotic or occluded, part of the blood from the vertebral artery can enter the ophthalmic artery through the Willis’ artery circle to make up the blood supply of the retina. The blood flow to the retina is reduced but not completely blocked. The retina is in a long-term hypoperfusion state, which is similar to the process of OIS^14^. Many studies on ocular ischemic diseases rely on acute ischemia–reperfusion models, but there are some limitations in mimicking chronic ischemia. Animal models of carotid artery occlusion were first established by Slakter *et al*. It was then found that BCCAO can lead to incomplete retinal ischemia and neuronal degeneration^15^. The chronic retinal ischemia model induced BCCAO complements the acute ischemia model. This model is the basis for the study of ocular ischemia, which is typically due to chronic hypoperfusion injury of the retina.

FFA is one of the most important diagnostic criteria for patients with OIS. About 95% of patients have prolonged arm-retina cycle times, which is the most specific FFA manifestation of OIS^5,16^. Our results show that the retinal A-V cycle in sham rats is approximately 2 seconds, whereas BCCAO rats had more severe defects in retinal venous circulation than in arteries, where the A-V cycle lasted for 10–30 seconds. Even at 1 minute, the veins could not be completely visualized. This study objectively observed the prolongation of rat tail–retina circulation time after BCCAO with retinal small hemangiomas and hemorrhages, venous dilation, and the non-perfusion zones, which is in line with the fundus features of patients with OIS^3,17^.

ERG is a commonly used method for the assessment of retinal nerve cells function. The a-wave represents the retinal photoreceptor function, and the b-wave represents the bipolar and Müller cell functions. According to other previous studies, in acute and chronic retinal ischemic, a decrease in the amplitudes of the a- and b-waves of ERG and a prolongation of the latency can be observed^18,19^. We found that under the same light stimulation condition, the latency of a- and b-waves in our BCCAO rats was significantly delayed and the amplitude was significantly reduced. This trend was aggravated by an increase in the hypoperfusion time. Unlike previous reports, the decrease of b-wave amplitude in the BCCAO rats is more significant than that of the a-wave, and it cannot return to the baseline level. This observation will be further confirmed and investigated in future studies.

There are still some difficulties in the monitoring of blood flow perfusion in clinical patients, but this can be compensated by animal experiments. The laser speckle blood flow imaging technology is a method for obtaining information on blood flow perfusion according to the backward dynamic speckle contrast value generated by red blood cell movement in blood vessels when the laser light is focused on the tissue surface. Owing to the advantages of noninvasive andc high-resolution imaging, it has been widely used to study the microcirculation at the body surface^20^. Some researchers have also used laser speckle imaging to measure microcirculatory changes in rabbit branch retinal vein occlusion (BRVO) models and rat cerebral infarction models^21,22^. The eye of the rat is small and its diopter is high. The laser cannot scan the blood vessels of the ocular fundus visually. Therefore ocular surface perfusion is used to evaluate the degree of ocular ischemia in BCCAO rats. The results show that as the time of ischemia increases, blood flow at the eyes’ surface of the model rats continued to decrease. To our knowledge, this is the first time that laser speckle imaging has been applied to the ocular ischemia model induced by BCCAO.

The most common pathologic manifestation of hypoperfusion retinopathy is retinal nerve cells apoptosis and glial activation hyperplasia^23^. The morphologic findings in this study showed that the thickness and structure of each retinal layer changed significantly and that RGCs began to present obvious apoptosis on the first week of BCCAO. On the second week, the apoptosis of the inner and outer nuclear layers of the retina aggravated, the nucleus dissolved, and vacuolar degeneration increased. At 4-week of BCCAO, the retinal total layer, inner nuclear layer and outer nuclear layer were thinnest due to atrophy, the nuclear membranes were shrinking and invaginated, chromatin density was uneven, and the mitochondria and other organelles were reduced. Consistent with previous research^12,24^, we compared the observations of different hypoperfusion stages more systematically. They can better reflect the conditions of OIS with different degrees of ischemia.

Taken together, our study suggested that BCCAO can induced retinal hypoperfusion injury in the model rats. The main manifestations were decreased blood perfusion, prolonged retinal circulation time, dysfunction of nerve cells conduction, and neuronal apoptosis at various levels of the retina. With increased ischemic time, the lesion showed progressive worsening. BCCAO could be used as an ideal animal model for the study of chronic ischemia in the eye. It will provide an experimental basis for further investigation of the pathogenesis and therapeutic measures of retinal hypoperfusion injury.

## Methods

### Animals

Sixty healthy adult male Sprague-Dawley rats, aged 4–6 weeks old and weighing about 200 g each were provided by the Beijing Huafukang Biotechnology Co., Ltd. (SCXK [Beijing] 2014–0004). The rats were bred at the SPF Animal Laboratory of China-Japan Friendship Hospital and the laboratory environment and facilities met the requirements of the national standard (GB 14925-2010). Breeding conditions were as follows: room temperature, 20–23 °C; relative humidity, 45–50%; light/dark cycle, 12/12 hours. All experimental operations were approved by the Animal Welfare Ethics Committee of China-Japan Friendship Hospital (Ethics No. 170208). All animals were maintained and handled in accordance with the ARVO Statement for the Use of Animals in Ophthalmic and Vision Research and the Declaration of Helsinki.

### Grouping and rat model of retinal hypoperfusion injury

**Figure 6:**
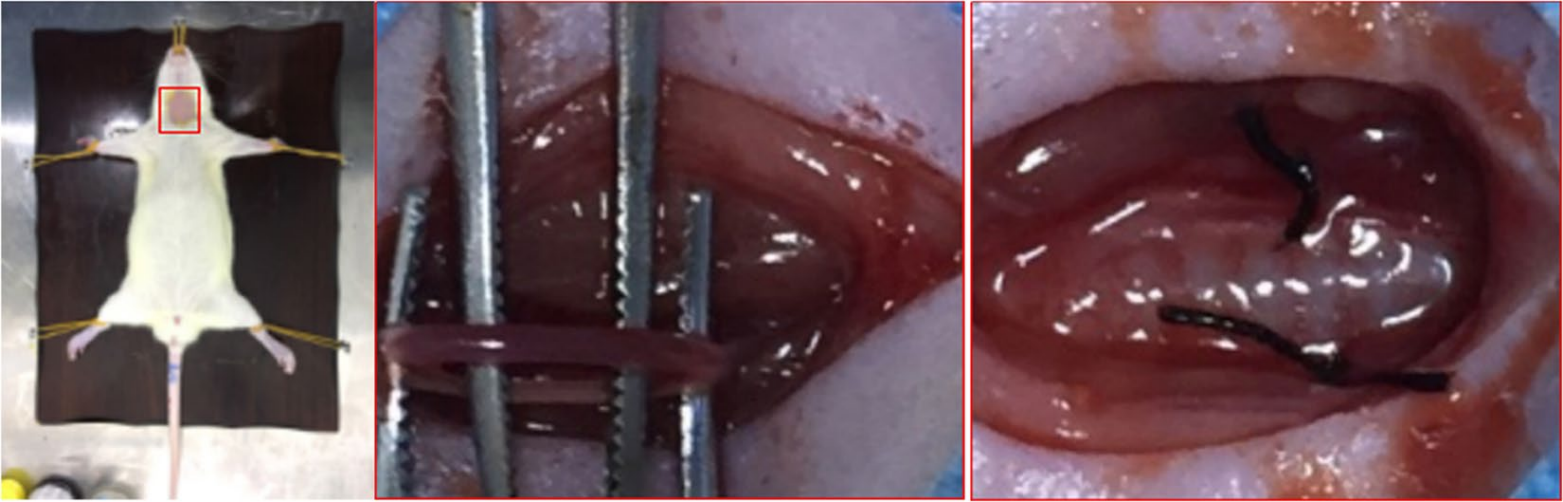
Model of bilateral common carotid artery occlusion. Bilateral carotid arteries of model rats were bluntly separated with vagus nerves and then fastened with 5–0 silk sutures.

The 60 rats were randomly divided into a sham surgery group (Sham, 30 rats) and a model group (BCCAO, 30 rats). All rats were fasted for 8–12 hours before surgery. After anesthesia induced by an intraperitoneal injection of 1% pentobarbital (50 mg/kg) (Sigma, American), each rat was placed in supine position and fixed on the operating table. The middle of the neck was sterilized and a 2–3 cm longitudinal incision along the midline mas made. Following sequential cutting of the skin, subcutaneous tissues, muscle layer, and fascia, bilateral carotid arteries of model rats were bluntly separated with vagus nerves and then fastened with 5–0 silk sutures (Fig. 6). 2–0 silk sutures were used to suture the muscle and skin. Ampicillin sodium (Solarbio, Beijing) was given intramuscularly (50 mg/d) for 3 days to prevent infection. The bilateral carotid arteries of the sham surgery rats were isolated without occlusion. Physiological parameters of rats in both groups were monitored before and after operation. The rats in the above two groups were randomly divided into 1, 2 and 4 weeks of three time subgroups.

### Fundus photograph and fluorescein fundus angiography

Tail-retinal blood circulation time were performed respectively by FFA at the 1, 2 and 4 weeks after the operation. After fully dilating the pupils, each rat’s head was fixed in front of a contrast machine (TOPCON TRC-501X, Japan). The lens focal length and camera exposure times were adjusted to take color photographs of the fundus and no-red light images. The rats’ tail skin was sterilized and the 10% fluorescein sodium (Alcon, Fort Worth, TX, USA) was injected into the dorsal vein of the tail (40 mg/kg) at once. Blood flow was confirmed before the syringe needle was fixed with tape. The operator began timing and the assistant quickly pushed the sodium fluorescein injection. The recording frequency was set at 1 picture per second with a 30° field range and exposure level at 50, centered on the disc, to obtain the center or other quadrant images. The blood circulation time of the retinal arteries and veins in each rat was recorded.

### Scotopic electroretinogram

**Figure 7:**
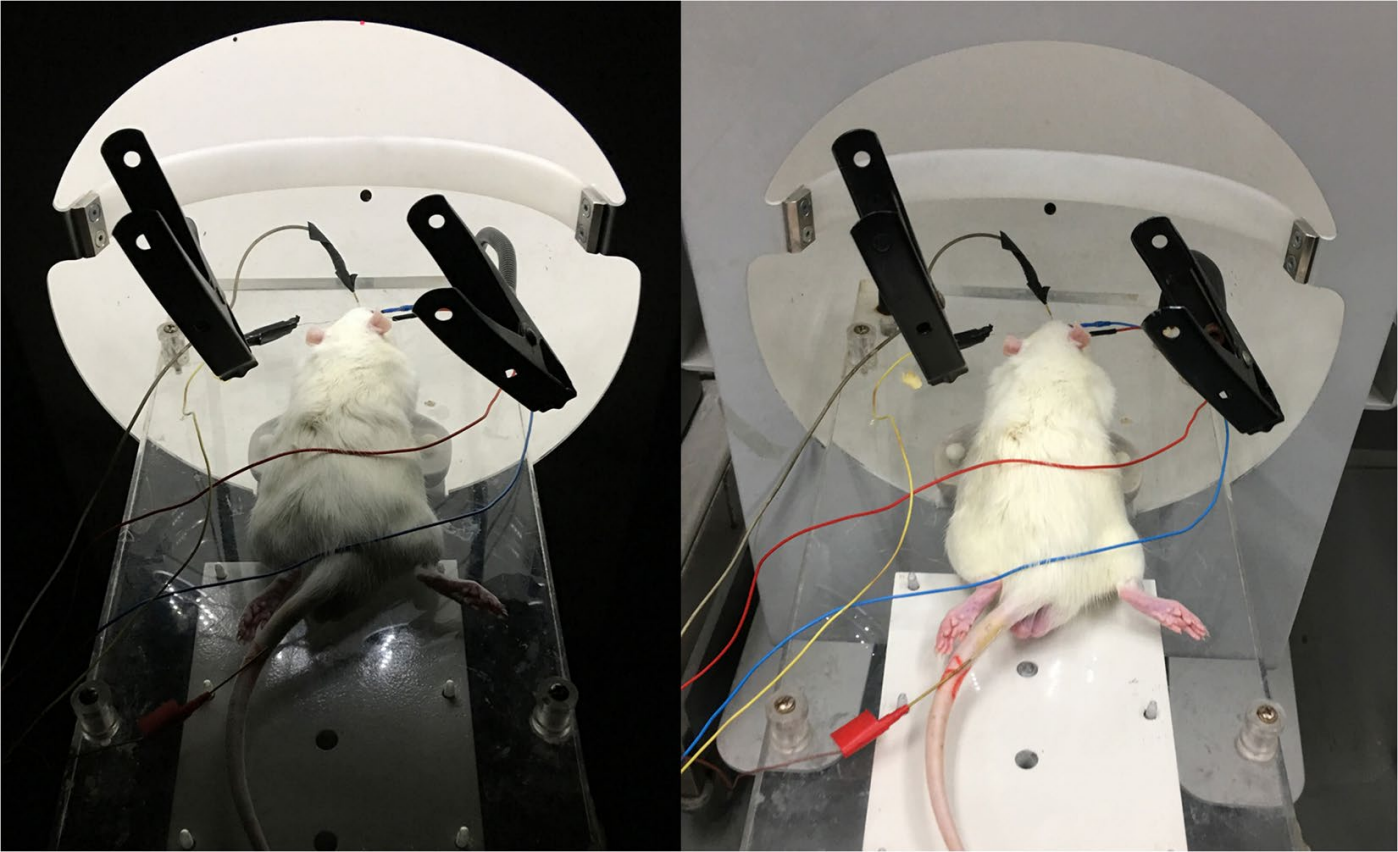
Electrodes connection of scotopic ERG. The left was a scotopic ERG check operation of rat, and the right with stopped operation was for clearer rendering.

In order to estimate the function of retinal nerve cells, scotopic ERG is usually recorded in the rats. Before examination, both groups of rats were placed in a dark room for dark adaptation for more than 2 hours. All steps must be performed under infrared light (Fig. 7). The pupils were fully dilated and the surface of the eyeball was anesthetized. Apply suitable amount of Vidisic to the surface of the cornea. The recording electrodes were fixed on the bilateral corneal surfaces; the reference electrodes were pierced into the rats’ bilateral cheeks; and the grounding electrode was placed subcutaneously into the tail base of each rat. Scotopic ERG was performed using the RET Iport system (Roland Consult, Germany) with a LED flash intensity white of -0dB (3.00 cds/m^2^), a stimulus frequency of 0.15 Hz, and a plot time of 150 ms. Recorded were changes of the latency (ms) and amplitude (μV) in a-wave and b-wave.

### Blood flow perfusion at the eye’s surface

During the experiments, we followed the strict operation room requirements. The constant temperature was setted about 23.0 + 1.0 °C and the relative humidity was about 55.0 + 5.0%. There was no direct sunlight, no obvious air convection and no noise interference. The rats in the two groups were given peritoneal anesthesia and placed on the operating board. The head of the laser speckle blood flow imager (PeriCam, PSI-NR, Sweden) was turned to the one side of the rat’s eyes. An effective sampling frequency of 1 image per second, a monitoring distance of 15 cm, a range of 5 × 5 cm, and a scanning time of 5 minutes were set for the blood flow perfusion of eye’s surface. Then switch to the other side of the rat’s eyes. The blood flow images of each rat were recorded, and the average blood flow perfusion (PU) of right and left eye was analyzed using PIM Soft blood flow imaging processing system. The blood flow images in each rat were defined as the area of interest (ROI) and the measured area of the eye’s surface was within 10 mm in diameter. The measured data were used for statistical analysis.

### Histological examination

Respectively at the 1, 2 and 4 weeks after the blood flow examinations, rats were sacrificed under isoflurane anesthesia. The eyes were immediately dissected in ice-cold phosphate buffered saline and fixed in 4% paraformaldehyde (Solarbio, Beijing). The anterior segment of the eyes and the vitreous were removed and the eyecups were left in 4% paraformaldehyde and fixed at 4 °C for 24 hours. Gradient alcohol was used for dehydration, xylene for clearing the sections, and paraffin for embedding. The retina was sectioned longitudinally at a thickness of 4 μm and then stained with hematoxylin-eosin (HE). The retinal histology and morphology was observed and photographed with microphotographic system (OLYMPUS BH-2, Japan). Image pro plus (IPP) software was used to measure the thickness of retinal total layer (RTL), inner nuclear layer (INL) and outer nuclear layer (ONL). The eyecups of rats in the 4 weeks were fixed in 2.5% glutaraldehyde solution (Solarbio, Beijing) and fully fixed at 4 °C for more than 72 hours. 1% citric acid was used to postfix for 1–2 hours and then washed with double distilled water twice, ethanol and acetone dehydration, Epon812 epoxy resin penetration, and embedded. The retina at 1 × 1 mm in size was used to make ultrathin slices at a thickness of 10 nm. Then 2% acetic acid uranyl and lead citrate were used to double stain the sections. The samples were observed and photographed under a Hitachi H-600 transmission electron microscope.

### Statistical analysis

Results were analyzed using SPSS Statistics 19.0 software. Comparisons of data between sham and model groups were performed using independent sample t- tests or t′ - tests. The results were expressed as mean ± standard deviation ($\overline{{\rm{X}}}$ ± s). A value of *P* < 0.05 represented statistical significance of differences between groups.

### Ethics approval

All experimental operations were reviewed by the Animal Welfare Ethics Committee of China-Japan Friendship Hospital (Ethics No. 170208).

## Electronic supplementary material

Supplementary information

## Data Availability

The datasets used and/or analysed during the current study are available from the corresponding author on reasonable request at any time.
