# Supplementary material for: Functional and morphologic study of retinal hypoperfusion injury induced by bilateral common carotid artery occlusion in rats
Source: Sci Rep. 2019 Jan 14;9:80. doi: 10.1038/s41598-018-36400-5 (PMC6331588; doi:10.1038/s41598-018-36400-5)
Supplement: Supplementary file 1 — Supplementary information [file 41598_2018_36400_MOESM1_ESM.pdf]

Title page

Title of the manuscript: Functional and morphologic study of retinal hypoperfusion injury induced by bilateral common carotid artery occlusion in rats

The authors list and email addresses:

Yali Qin[1,2], qinyali163@163.com

Meiqi Ji[1,2], jimeiqi9966@sina.com

Tingting Deng[3], ttdeng1983@163.com

Dan Luo[2], cia100@sina.com

Yingxin Zi[1,2], ziyingxin@126.com

Lin Pan[3], panl2005@163.com

Zhijun Wang[2], wangzj301@sina.com

Ming Jin[2], jinming57@163.com

[1]Beijing University of Chinese Medicine, Beijing, 100029, China.

[2]Department of Ophthalmology, China-Japan Friendship Hospital, Beijing, 100029, China.

[3]Clinical Medical Research Institute, China-Japan Friendship Hospital, Beijing 100029, China.

Correspondence: M. J. (email: jinming57@163.com).

Supplementary information

Physiological parameters: Before and after model surgery, the blood pressure and pulse of rats were monitored by BP-2000 Animal Non-invasive Blood Pressure Analyzer (Beijing Mingxintong Biological Technology Co., LTD). The rats were put into the thermostats of the blood pressure instrument to measure the tails' artery systolic blood pressure (SBP) and pulse in awaking and quiet conditon of the rats, and the average result was obtained after three automatic tests. And the changes of blood oxygen saturation (SO2) in the microvessels of the rats forehead were detected by the German O2Ceva Time Easy Examination for animals before and after model surgery. Centered on the intersection of the rats cranial coronal suture with left and right temporal bone, the scalp was incised with 5mm in diameter and the detection time was 10 seconds. The outcomes showed that there was no obvious change in the tails' artery systolic blood pressure of the model group rats before and after BCCAO ($P >$ 0.05), but the blood oxygen saturation of model rats after BCCAO was significantly decreased (P <0.01).

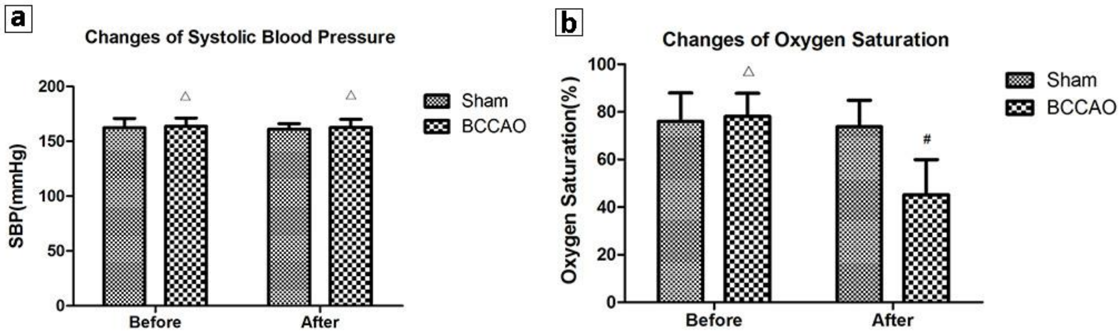

Physiological parameters of rats before and after model surgery. (a) The changes of systolic systolic blood pressure (SBP). $^{\triangle}P > 0.05$: there was no obvious change in the tails' artery systolic blood pressure and pulse of the model group rats before and after BCCAO. (b) The changes of oxygen saturation (SO_2). $^{\#}P$ <0.01: compared with before surgery in model group and sham group, the blood oxygen saturation of model rats after BCCAO was significantly decreased.
